# Primary cardiac angiosarcoma diagnosed in the first trimester of pregnancy

**DOI:** 10.3332/ecancer.2019.922

**Published:** 2019-04-09

**Authors:** Ethan A Burns, Amna Ahmed, Anusha Sunkara, Usman Khan, Roozbeh Sharif, Maen Abdelrahim, Michael Reardon, Barry Trachtenberg

**Affiliations:** 1 Internal Medicine, Houston Methodist Hospital, Houston, TX 77030, USA; 2 Houston Methodist DeBakey Heart and Vascular Center, Houston, TX 77030, USA; 3 Houston Methodist Cancer Center, Houston, TX 77030, USA; 4 Houston Methodist Critical Care, Houston, TX 77030, USA

**Keywords:** pregnancy, pericardial tamponade, primary cardiac angiosarcoma, pregnancy, echocardiography, cardiac magnetic resonance imaging, chemotherapy

## Abstract

Primary cardiac angiosarcoma (PCAS) is a malignancy seldom seen in pregnancy. A 23-year-old G1P0 Chinese female was found to have PCAS during her first trimester when she presented with tamponade physiology. The transthoracic echocardiography (TTE) results did not indicate the presence of an intracardiac lesion, and pericardial fluid cytology analysis showed no evidence of malignancy. Cardiac magnetic resonance imaging (CMRI) exhibited a right atrial mass, and tissue biopsy indicated a high-grade angiosarcoma. MRI of the abdomen was suggestive of liver metastasis. She underwent an abortion and was started on combination chemotherapy, with a reduction in both the cardiac and liver masses. In cardiac angiosarcomas, advanced imaging modalities such as MRI should be utilised when there is high clinical suspicion or in the case of pregnancy when trying to minimise foetal harm. Prognosis is poor, and a standardised treatment protocol regardless of pregnancy continues to elude the medical community.

## Introduction

Angiosarcoma is a rapidly proliferating malignant neoplasm derived from anaplastic endothelial cells. It most commonly arises from the soft tissues of the head, neck, breast, liver, skin and deep tissue, and is clinically unpredictable [1, 2]. It comprises 1% of all sarcomas which is less than 1% of solid malignancies [1, 3]. This is an aggressive neoplasm with a high rate of recurrence, irrespective of treatment modality.

Primary cardiac tumours are exceedingly rare, with an estimated incidence of 0.001%–0.03% [4]. Approximately 25% are malignant, and the majority are primary cardiac angiosarcoma (PCAS) [5]. The co-occurrence of PCAS and pregnancy is seldom reported [6–13]. We present a case of PCAS in a pregnant patient which is associated with diagnostic and treatment challenges.

## Case

A 23-year-old previously healthy Chinese female who was 4 weeks gravid at admission presented with 2 weeks of nausea, vomiting, abdominal pain and chest pain. Her chest pain was sharp and retrosternal, unrelated to activity, and did not vary with position or respiration.

On admission, she was tachycardic but otherwise haemodynamically stable. Physical exam was notable for decreased heart sounds, abdominal distention and ascites. Electrocardiogram (EKG) findings demonstrated sinus tachycardia and low voltage QRS. Labs were significant for hyponatremia (131 mEq/L), anion gap metabolic acidosis, elevated International Normalized Ratio (INR) (1.8), Aspartate Aminotransferase (AST) (64 U/L) and Alanine Aminotransferase (ALT) (58 U/L). Chest x-ray revealed bilateral pleural effusions, and abdominal ultrasound showed hyperechoic masses on the liver.

**Figure 1.**
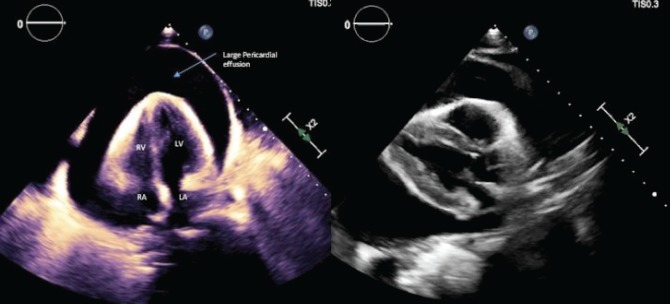
(a) Transthoracic echocardiogram, subcoastal view with a large pericardial effusion. (b) Transthoracic echocardiogram, parasternal long-axis view demonstrating a large pericardial effusion.

**Figure 2.**
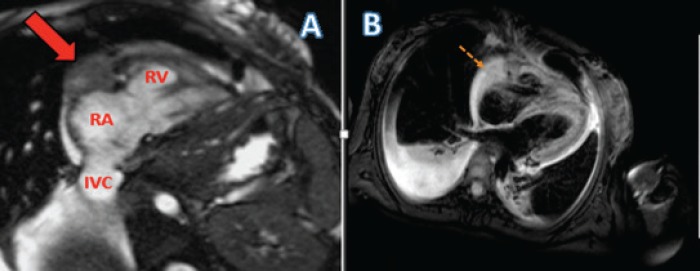
(a) Cardiac MRI of the patient with cine RV-2 chamber view showing the pericardial mass encasing the right atrioventricular groove (AV) groove (solid red arrow). Labelled RA: Right atrium; RV: Right ventricle; IVC: Inferior vena cava. (b) Tissue characteristics of the right atrial mass.

**Figure 3.**
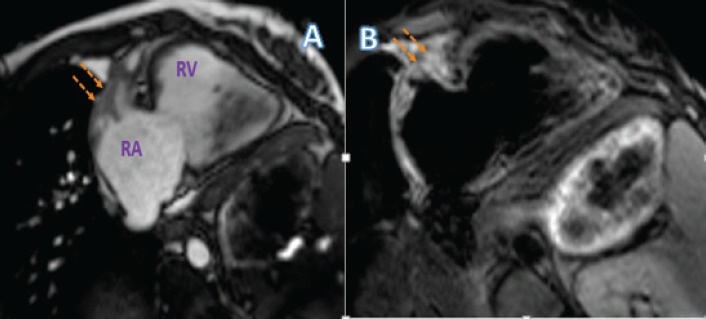
(a) Cardiac MRI of the patient post two cycles of chemotherapy with cine RV-2 chamber view showing the reduced pericardial mass encasing the right AV groove. (b) Tissue characteristics of the right atrial mass.

Paracentesis of the ascitic fluid suggested a transudative process. Abdominal magnetic resonance imaging (MRI) demonstrated a 3.7 cm mass in the right hepatic lobe with decreased signal intensity on T1 and numerous hyperintense nodules on T2-weighted imaging concerning for metastatic disease. Marked cardiomegaly was incidentally noted. Liver biopsy showed anastomosing hemangiomas without evidence of malignancy. During workup, she developed hypotension, tachycardia and dyspnoea. An emergent transthoracic echocardiography (TTE) showed a large pericardial effusion with early tamponade (Figure 1). She had an emergent pericardial window with mediastinal drain placement; cytopathologic and microbiologic analysis from pericardial fluid were negative for malignancy and infection. Two subsequent TTEs showed right ventricular systolic dysfunction, right atrial enlargement and an ejection fraction (EF) of 40%–44%. Due to the patient’s tamponade and worsening cardiac function without an identifiable cause, a cardiac MRI (CMRI) was done to better assess the patient’s cardiac structure and function (Figure 2). Her CMRI demonstrated a large, ill-defined right atrial mass isointense on T1 and hyperintense on T2 (Figures 2 and 3).

**Figure 4.**
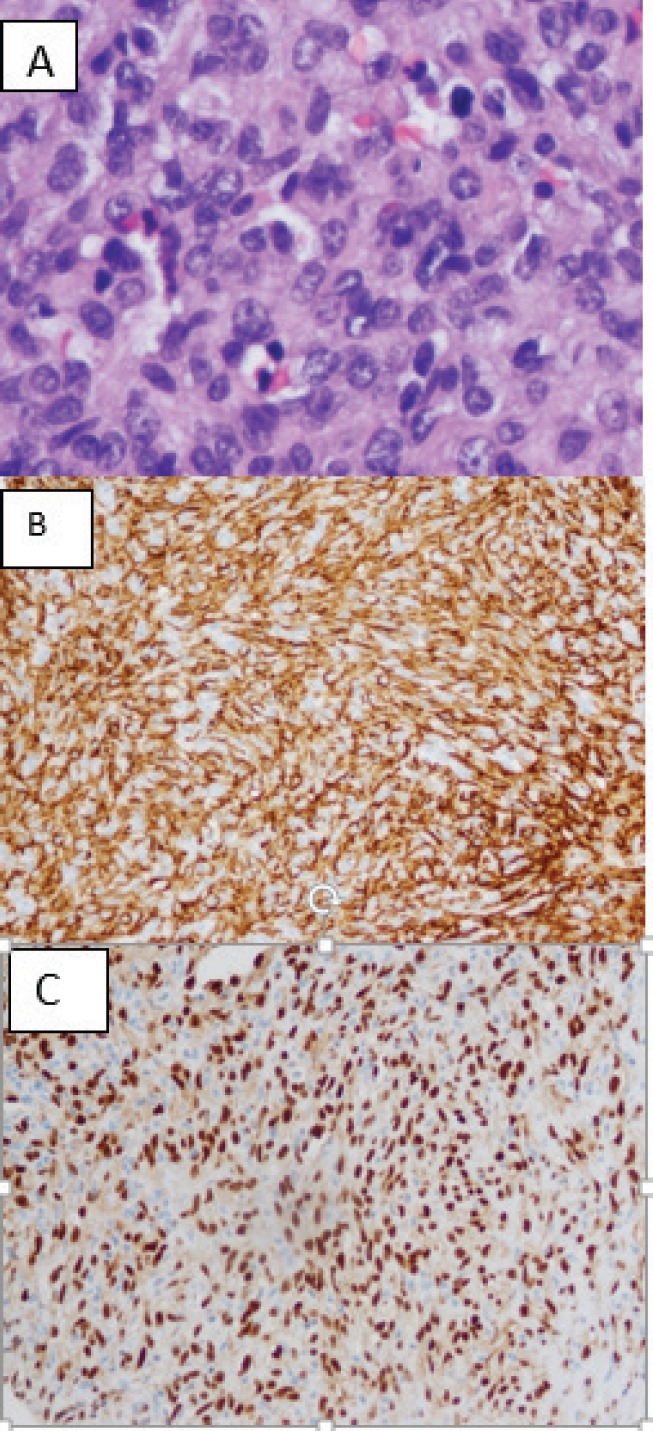
(a) H&E stain of right atrium cardiac biopsy of the patient showing high-grade spindle cell proliferation and hyperchromatic and pleomorphic nuclei with mitotic activity. (b) Immunohistochemistry of vascular differentiation markers, strongly positive for CD31. (c) Immunohistochemistry of vascular differentiation markers, strongly positive ETS (Erythroblast transformation specific) related gene.

The patient had an open biopsy of the cardiac mass via a right anterior thoracotomy approach that indicated a high-grade PCAS (Figure 4). Staging workup with Positron Emission Tomography (PET)-CT showed increased uptake in the right atrial mass. Despite a non-diagnostic liver biopsy for metastasis, imaging raised concern for metastatic disease and the patient was treated as such. The patient opted for elective termination of pregnancy and was started on combination adriamycin and ifosfamide chemotherapy. Her EF dropped by 10% after completing two cycles of therapy and was subsequently switched to a second-line regimen with gemcitabine and docetaxel chemotherapy. Repeat staging 4 months later showed absent Fluorodeoxyglucose (FDG) uptake in the right atrium and decreased tumour burden (1.3 cm × 1.0 cm) (Figure 2) along with a decreased size of the hepatic lesions. Nine months after initial diagnosis, she continues to receive chemotherapy without disease progression.

## Discussion

PCAS is a malignancy that is seldom documented. There is a male-to-female predominance (2:1 ratio) and it occurs between the third and fifth decades of life [14, 15]. There are a few documented cases describing the co-occurrence of PCAS and pregnancy between 1969 and 2015, with all cases occurring in the latter half of pregnancy [6–13]. This is the first known report of PCAS diagnosed during the first trimester.

PCAS commonly arises from the right atrial free wall [16]. Symptoms manifest secondary to the obstruction of blood flow, myocardial invasion and metastatic spread; this results in arrhythmias, pericardial and pleural effusions and embolisation [17]. Many individuals have metastatic disease when symptoms manifest [14, 18, 19] with the most common sites being the lungs, liver, lymph nodes, bone, adrenal glands and spleen [17]. The symptoms in this patient were initially attributed to pregnancy. However, the abdominal distension out of proportion to her 4-week gestational age combined with the decreased heart and breath sounds raised concern for an underlying pathologic process. Symptoms concerning an underlying cardiovascular aetiology in pregnancy should always prompt further investigation. In addition, PCAS should be considered in the differential diagnosis in pregnant women presenting with cardiac tamponade and should have further diagnostic workup to rule out this aggressive and rapidly fatal malignancy.

Early diagnosis of PCAS is clinically difficult. Pregnancy further complicates workup due to the teratogenic, carcinogenic and mutagenic risk associated with certain imaging modalities on the developing foetus. TTE often shows a broad-based mass [20] though no intracardiac masses were seen on three different TTEs in this case. Multi-slice computed tomography (MSCT) shows a low attenuated mass and can assess the degree of tumour burden and metastatic spread and is useful for distinguishing between cardiac thrombus and tumour [21, 22]. Transesophageal echocardiography (TEE) can assess malignant potential by determining the degree of local invasion [21]. Advanced cardiac imaging modalities such as gated cardiac computed tomography (Cardiac CT) and CMRI are often utilised when cardiac tumours are suspected. CMRI may demonstrate heterogeneous signal intensity, with the isointense signal on T1-weighted imaging and hyperintensity on T2-weighted imaging [23]. Furthermore, PCAS may have more specific findings such as a papillary appearance on CMRI or a ‘sunray’ appearance when diffuse pericardial infiltration is present [24]. Cardiac CT shows a broad-based, heterogeneous mass with low attenuation, and is useful for assessing local infiltration [22].

Imaging modalities should be utilised with caution in any pregnant female wishing to carry their pregnancy to term, and a multidisciplinary discussion regarding a cancer staging workup should be considered. When determining imaging modalities for staging, safety of the foetus, likelihood of metastatic disease and the minimal radiation dose to achieve optimal staging accuracy should be considered [25]. With regard to ionising radiation, the concentration of foetal exposure may be 8–25 mGy with CT of the abdomen and pelvis, 0.01–0.66 mGy with CT of the chest and 1.1–9.04 mGy with FDG-PET [25, 26]. In the first two trimesters, exposure to greater than 50–100 mGy can predispose the developing foetus to mental retardation, microcephaly, intrauterine growth retardation and increased teratogenicity [26]. Clinicians should be mindful of this when considering initial imaging and serial staging. When considering MRI, there is a theoretical risk that the magnetic field can lead to alterations in cellular migration, proliferation and differentiation; as a result, the International Commission on Non-Iodizing Radiation Protection recommends postponing elective MRI until after the first trimester [27, 28]. In 2013, the expert panel on MRI safety practices stated that MRI can be obtained regardless of gestational age when the information gained is likely to alter treatment, cannot be obtained through other nonionising means and when it cannot be delayed through the completion of pregnancy [25, 29].

As the patient in this case opted for elective abortion, radiation exposure to the foetus was not a limiting factor. Regardless, CMRI was indicated to assess the patient’s cardiac structure and function, given her unprovoked cardiac tamponade and progressive heart failure seen on serial TTEs [30].

If imaging is suggestive of a cardiac mass that is concerning for malignancy, further workup with cytology and tissue biopsy is necessary to confirm the diagnosis. Pericardial fluid cytology has a sensitivity of 87%–100%, specificity of 93.3%–100% and diagnostic accuracy of 94%–95.4% [31, 32]. However, cytology may not always be suggestive of malignancy, as this case demonstrated. Gross examination commonly reveals haemorrhage and necrosis. Histology demonstrates anastomosing vascular channels and poorly differentiated spindle and epithelioid cells with frequent mitosis [33]. Immunohistochemistry is an adjunctive diagnostic tool to delineate the endothelial origins of PCAS from other neoplasms. CD31, a transmembrane glycoprotein, friend leukaemia virus integration-1 (FLI-1), a transcription factor in vascular tumours, and CD34, a hematopoietic progenitor cell antigen, are often seen in PCAS [33]. Immunohistochemical stains have implicated overexpression of vascular endothelial growth factors, protein kinases A and C, as well as p-AKT/mTOR in the pathogenesis of PCAS [34], though it is uncertain if pregnancy impacts these cascades.

The prognosis for PCAS is poor, with a median survival of 6–30 months [14, 33, 35]. When diagnosed at an early stage, radical resection is recommended to prolong survival. However, surgery is rarely curative due to the proximity of other structures and the degree of local invasion [8, 14]. In the rare co-occurrence of pregnancy and PCAS, surgery offers a palliative approach that may allow pregnancy to reach foetal viability. Adjuvant chemotherapy is an additional option but may pose a significant risk to the foetus. Chemotherapy during the first trimester carries the highest risk for foetal teratogenicity and death although a study by Aviles et al [36]. indicated that cytotoxic chemotherapy used in the first trimester may not always lead to major foetal complications [37]. Chemotherapy in the second and third trimesters typically has little effect on the developing foetus and long-term outcomes on the child [38], and thus certain agents can be considered in the treatment of PCAS while allowing the pregnancy to progress to term. Commonly utilised chemotherapeutic agents include cisplatin, cyclophosphamide, dacarbazine, adriamycin, ifosfamide, mitomycin-C, paclitaxel and vincristine [35]. Immunotherapy has been utilised, with studies demonstrating a 30-month median survival following combination of surgery, chemotherapy and recombinant IL-2 therapy [39, 40]. A combination treatment modality is favoured though a standardised algorithm has not been determined.

The anatomic location of PCAS often dictates management. Right-sided PCAS are bulky, infiltrative and rapidly metastasise. Alternatively, left-sided PCAS are well circumscribed and are more often associated with heart failure [41]. In the cohort of patients with right-sided PCAS amenable to resection, Blackmon and Reardon had success with neoadjuvant chemotherapy to maximise tumour shrinking prior to resection [41]. Furthermore, Abu Saleh et al. demonstrated a doubling of survival time (20 versus 9.5 months) with neoadjuvant chemotherapy in patients with right-sided PCAS, with further survival benefit when neoadjuvant therapy led to R0 margins prior to surgical resection (53.5 versus 9.5 months for R1) [42]. As left-sided PCAS is frequently associated with heart failure that could be exacerbated with the use of chemotherapeutic agents, neoadjuvant chemotherapy is not recommended [39]. Cardiac transplantation has been suggested as a treatment modality in high-grade PCAS in younger individuals, but studies have not demonstrated a survival benefit [43].

## Conclusion

PCAS is a rare malignancy and this is the first known case of PCAS diagnosed in a first-trimester pregnant patient. PCAS should be on the differential diagnosis in pregnant females presenting with tamponade or heart failure symptoms. If foetal viability is the goal, imaging modalities should be chosen carefully to limit harm. Routine imaging modalities such as TTE may not always detect the presence of intracardiac tumours, as this case suggests. Pericardial fluid cytology has high sensitivity and specificity but may miss the diagnosis as demonstrated in this case. Advanced imaging modalities such as CMRI have excellent diagnostic yield and can demonstrate findings specific to PCAS. Although there is a theoretical risk impacting cellular development, this has not been substantiated in humans. Treatment in the pregnant patient is often a palliative surgical approach if detected at an early stage to let the foetus mature to a viable age. Chemotherapy puts the foetus at high risk of harm if used in the first trimester, but certain agents can be used in the second/third trimester with minimal risk to the developing foetus. If chemotherapy is used, combination chemotherapy is favoured. Overall prognosis remains poor, and it is unknown if pregnancy impacts mortality or leads to accelerated pathogenesis compared to the nonpregnant population.

## Conflicts of interest

The authors declare that they have no conflicts of interest.

## Funding declaration

The authors received no specific funding for this work.
